# Phenotype and mutation expansion of the *PTPN23* associated disorder characterized by neurodevelopmental delay and structural brain abnormalities

**DOI:** 10.1038/s41431-019-0487-1

**Published:** 2019-08-08

**Authors:** Renee Bend, Lior Cohen, Melissa T. Carter, Michael J. Lyons, Dmitriy Niyazov, Mohamad A. Mikati, Samantha K. Rojas, Richard E. Person, Yue Si, Ingrid M. Wentzensen, Regeneron Genetics Center, Erin Torti, Jennifer A. Lee, Kym M. Boycott, Lina Basel-Salmon, Carlos R. Ferreira, Claudia Gonzaga-Jauregui

**Affiliations:** 1 Greenwood Genetic Center, Greenwood, SC 29646 USA; 2 Raphael Recanati Genetic Institute, Schneider Children’s Medical Center of Israel, 49100 Petah-Tiqva, Israel; 3 Children’s Hospital of Eastern Ontario Research Institute, University of Ottawa, Ottawa, ON K1H 8L1 Canada; 4 Ochsner Medical Center, New Orleans, LA 70121 USA; 5 Duke Institute for Brain Sciences, Durham, NC 27710 USA; 6 GeneDx, Gaithersburg, MD 20877 USA; 7 Children’s National Medical Center, Washington, DC 20010 USA; 8 Regeneron Genetics Center, Regeneron Pharmaceuticals Inc., Tarrytown, NY 10599 USA

**Keywords:** Neurodevelopmental disorders, Medical genetics, Mutation, DNA sequencing

## Abstract

*PTPN23* is a His-domain protein-tyrosine phosphatase implicated in ciliogenesis, the endosomal sorting complex required for transport (ESCRT) pathway, and RNA splicing. Until recently, no defined human phenotype had been associated with alterations in this gene. We identified and report a cohort of seven patients with either homozygous or compound heterozygous rare deleterious variants in *PTPN23*. Combined with four patients previously reported, a total of 11 patients with this disorder have now been identified. We expand the phenotypic and variation spectrum associated with defects in this gene. Patients have strong phenotypic overlap, suggesting a defined autosomal recessive syndrome caused by reduced function of *PTPN23*. Shared characteristics of affected individuals include developmental delay, brain abnormalities (mainly ventriculomegaly and/or brain atrophy), intellectual disability, spasticity, language disorder, microcephaly, optic atrophy, and seizures. We observe a broad range of variants across patients that are likely strongly reducing the expression or disrupting the function of the protein. However, we do not observe any patients with an allele combination predicted to result in complete loss of function of *PTPN23*, as this is likely incompatible with life, consistent with reported embryonic lethality in the mouse. None of the observed or reported variants are recurrent, although some have been identified in homozygosis in patients from consanguineous populations. This study expands the phenotypic and molecular spectrum of *PTPN23* associated disease and identifies major shared features among patients affected with this disorder, while providing additional support to the important role of *PTPN23* in human nervous and visual system development and function.

## Introduction

The wider application of genomic sequencing technologies, either through whole genome or whole exome sequencing (WES), has enabled the study and molecular characterization of many disorders not previously described. Genomic sequencing of cohorts of patients with shared major clinical features, such as developmental disorders [1], intellectual disability and developmental delay, brain malformations [2], congenital heart disease [3], or pulmonary hypertension [4] has successfully identified causative genes, while at the same time demonstrating the genetic heterogeneity of these broadly defined disorders. Furthermore, novel genes associated with such disorders may be identified in these cohorts; however, burden of evidence can be challenging for new candidate genes if only one patient is identified with potentially disease causing variants in a yet unreported disease gene. Ideally, evidence in support of a novel gene-disease association can be attained by identifying additional patients with overlapping clinical features and the same or functionally similar variants in the novel candidate disease gene, while further defining the phenotypic spectrum of such novel rare disorders.

*PTPN23* encodes the 1636 amino acid non-receptor protein tyrosine phosphatase type 23 that appears to be essential for endocytic trafficking. It interacts with ESCRT (endosomal sorting complex required for transport) complexes to sort ubiquitinated proteins into multi-vesicular bodies (MVBs) [5, 6]. Depletion of the protein has been shown to cause accumulation of ubiquitinated proteins in the endosomes and disrupt the morphogenesis of MVBs [6]. *PTPN23* has been proposed as a tumor suppressor gene due to its apparent role in cell migration and proposed role in negatively regulating RAS mediated mitotic proliferation through its catalytically inactive C-terminal protein-tyrosine phosphatase (PTP) domain, independently from any PTP activity [7–9]. Finally, it appears to be important for the endurance of the survival of motor neuron (SMN) complex by maintaining its phosphorylation [10]. In mice, *PTPN23* appears to be essential for embryogenesis, with embryonic lethality occurring at around E9.5, and significant abnormalities and smaller size observed at E8.5 in homozygous knock out embryos (*Ptpn23*^−*/−*^*)* [11]. During early embryonic development, it is predominantly expressed in the nervous system, especially in the mid and hindbrain, the optic and otic vesicles, and the ventricular layer of the forebrain. As development progresses, *PTPN23* expression becomes broader, with some detectable expression across most organs [9, 11]. In adult mice, *PTPN23* continues to be expressed in several brain regions including the cerebral cortex, the thalamus, and the hypothalamus [11]. In addition, it has been shown that the *Drosophila* orthologue of *PTPN23*, *Myopic (mop)*, is involved in neuronal specification and central nervous system development in this organism [12]. Myopic has been shown to regulate neuropeptide secretion in the neuromuscular junction of the fly by selectively participating in the exocytosis of neuropeptide containing synaptic dense-core vesicles (DCVs) without affecting the release of small-molecule neurotransmitters from small synaptic vesicles (SSVs) [13].

Until recently, protein altering variants in *PTPN23* had not been linked with any described human phenotype. In 2015, Alazami et al. reported *PTPN23* as one of 69 novel recessive candidate genes identified through WES of multiplex consanguineous families segregating neurogenetic disorders [14]. In 2016, Trujillano et al. identified a second patient with a rare homozygous variant in *PTPN23* within a cohort of 1000 families [15]. More recently, Sowada et al. [16] and Smigiel et al. [17], separately reported two unrelated affected female probands with compound heterozygous variants in this gene. Here, we report an additional seven patients with biallelic rare variants in *PTPN23* identified through WES analyses and multi-center international collaboration. The molecular and clinical characterization of this cohort confirms the gene-disease association of *PTPN23* with a severe neurodevelopmental disorder characterized by developmental delay and structural brain abnormalities, and expands the range of clinical features that may be observed in patients with this novel rare genetic disorder.

## Subjects and methods

### Informed consent

Patients and family members included in this report were consented for genetic and genomic studies, publication of clinical and genotype data, and photography through Institutional Review Board (IRB) approved protocols of the corresponding institutions. Patients are numbered according to their age at evaluation.

### Patients and molecular studies

Proband 1 was evaluated at Children’s National Medical Center (CNMC) in Washington, DC, USA. Previous testing included chromosomal microarray that revealed a 15q11.2 BP1-BP2 microdeletion (Table 1). This 512 kb deletion is common (seen in about 1% of patients in whom a microarray is obtained for neurological problems), has low penetrance of about 10% [18], and was deemed to possibly explain only his developmental delay, but not the rest of his multisystemic phenotype. Parental testing was not obtained, although it is known that ~50% of all cases with this microdeletion inherited it from a parent with no known health or learning problems [19]. Exome sequencing was performed through a commercial laboratory (Baylor Miraca Genetics Laboratories).

**Table 1:** Clinical characteristics of patients with biallelic protein altering variants in *PTPN23*

	Patient 1	Patient 2	Patient 3	Patient 4	Patient 5	Patient 6	Patient 7	Alazami, 2015	Trujillano, 2017	Sowada, 2017	Smigiel, 2018
Age at evaluation	14 months	3-year-old	6-year-old	7-year-old	10-year-old	11-year-old	13-year-old	5-year-old	NA	4-year-old	6-year-old
Sex	M	M	M	F	F	F	M	M	NA	F	F
Ethnicity	Kuwaiti	European	North American	Syrian	Ashkenazi Jewish	European	Hispanic	Saudi	NA	European	European
Variant zygosity	Comp Het	Comp Het	Comp Het	Homozygous	Homozygous	Homozygous	Comp Het	Homozygous	Homozygous	Comp Het	Comp Het
Variants	c.2680 C > T; p.His894Tyr | c.2747 A > G; p.Gln916Arg	c.3748 G > A; p.Glu1250Lys | c.2878_2889del12; p.Gln960_Pro963del	c.1291 C > T; p.Arg431Trp | c.2486 C > T; p.Pro829Leu	c.2568_2594del27; p.Val857_Pro865del	c.3884_3886delAGA; p.1295_1296delLys	c.4651_4652dup; p.Leu1552Hisfs*33	c.1748A > G; p.Lys583Arg, c.3051 G > C; p.Gln1017His| c.695 G > A; p.Arg232Gln	c.3995 G > T; p.Arg1332Leu	c.904 A > G; p.Met302Val	c.1595 C > T; p.Pro532Leu | c.3586 C > T; p.Arg1196*	c.1902C > T; p.Asn634Lys | c.2974delC; p.Leu992Tyrfs*168
Consanguinity	No	No	No	Yes	No	No	No	Yes	NA	No	No
Developmental delay	+	+	+	+; Profound, no milestones	+	+; Regression at 2 years old	+	+	+; regression	+	+
Intellectual disability	+	+	+	+	+; Mild	+; Cerebral palsy	+	+; cerebral palsy	+	+	+
Langiuage delay	+; Borderline	+	Dyspraxia	+; Absent language	+	+; No expressive language	+; Expressive language delay (receptive better), non-verbal	+; No Expressive language	+	NA	+; Absent language
Optic atrophy	0.35 c/d, anomalous looking optic nerve (OU)	NA	−	Significant visual impairment	+	+	−	NA	NA	+	+
Microcephaly	−	–	Borderline (10%ile)	+; 47 cm	−	+	−	+; Progressive	+	+	+
Seizures	−	-; Abnormal EEG	−	+; Febrile	−	−	+; Intractable seizures	+	+	+	+
Tone/movement disorder	NA	+; Tremor	+; Apraxia	+; Spasticity and contractures; wheelchair bound, little purposeful movement	−	+; Spastic diplegia, contractures	+; Areflexia, contractures, non-ambulatory	+; Spasticity	+; Spasticity	+; Spasticity	+; Spasticity
Abnormal MRI findings	*Ventriculomegaly*; mild *decrease in cerebral volume*	*Ventriculomegaly; cerebral volume loss*	Unremarkable brain MRI	Unavailable, *brain atrophy* and lissencephaly per parents report	Insular and parietoocipital polymicrogyria with *partial agenesis of corpus callosum, hypoplasia of optic nerves*	*Ventriculomegaly*; delayed myelination	Bilateral *brain atrophy*	*Ventriculomegaly*; severe *brain atrophy*; marked *loss of periventricular white matter*, mild cerebellar atrophy; markedly thinned corpus callosum, large cisterna magna, and mild vermis hypoplasia.	*Brain atrophy* unspecified	*Ventriculomegaly*; severe *brain volume reduction*; hypoplastic corpus callosum. Basal ganglia, brain stem, and cerebellum appeared normal. *Hypoplastic optic nerves* and optic discs	Abnormal myelination, progressive cortical-subcortical atrophy with *decreasing volume*, rarefaction of white matter, *widening of lateral ventricles*; cerebellar atrophy; *optic nerve atrophy*
Dysmorphic features	Brachycephaly, metopic ridging, left esotropia, arched eyebrows, mild micrognathia, deep palmar creases; plantar pads	Prominent forehead, deep set eyes, short palpebral fissures, prominent nasal root, smooth philtrum with thin upper lip, low set, protruding and simple ears	Synophrys, broad and flat nasal bridge, full lips	Long and narrow face with very prominent ears	Long face, prominent forehead,malar hypoplasia, long and smooth philtrum, full lips, broad and long mouth,hypoplasia of ala nasi,anteverted nares,bulbous nasal tip, collumela below nares, posterior rotated ears, narrow and high-arched palate, fetal fat pads	Strabismus, thin eyebrows, prominent and wide-spaced upper incisors; full lips	None noted	NA	NA	NA	Protruding tongue
Other findings	Tracheoesophageal fistula, mild left pelviectasis	Abnormal EEG (frequent isolated right parietal spike discharges) without overt seizures	VSD/PDA, autism, dyspraxia, hyperlexia, sensory processing disorder	Severe growth restriction/failure to thrive; cataract; recurrent infections/aspiration pneumonia; scoliosis; died at 7 years of age	Difficulty with social interactions; pes planus, no skeletal abnormalities; VEP- optic neuropathy	Femoral anteversion, constipation, hypotonia, hyperreflexia, slow gait, bent knees, narrow hands and feet.	Short stature, scoliosis, strabismus, hypotonia, precocious puberty, central sleep apnea, insomnia.	Gastroesophageal reflux disease, cortical blindness, wheelchair-bound, peripheral hypertonia. Patient died at 9 years of age of a chest infection.	Developmental regression	Severe encephalopathy with progressive spasticity and no cognitive and motor development at all; child died of pneumonia at age 4 years and 10 months	Hypotonia followed by spasticity; abnormal EEG showing multifocal discharges; hearing loss; central thyroid insufficiency
Previous testing	Chromosome microarray: arr[hg19] 15q11.2(22,770,421 to 23,282,905)x1	NA	Chromosome microarray, fragile X normal	Chromosome microarray normal; rare homozygous variant in *EOMES* identified in linkage with *PTPN23* variant within the same region of homozygosity	Normal karyotype and chromosome microarray testing; negative WBS	Chromosome microarray: 15q13.3 440.5 kb gain (VUS), and 14.3 Mb block of Absence of Heterozygosity (AOH) at 3p21.31p14.2; Rett syndrome and Prader Willi/Angelman testing normal	Amino acids, lactate, pyruvate and acylcarnitine profile, karyotype, thyroid function, muscular dystrophy testing, urine organic acids, FISH for Prader-Willi-Angelman	NA	NA	NA	Normal amino acids profile. Lactic acid, CK and NH3 levels normal. Normal karyotype, Angelman methylation and MLPA testing normal, *SETBP1* gene sequencing normal. Chromosome microarray: Xp22.33 756 kb gain inherited from unaffected mother

Probands 2 and 4 are from unrelated families and were evaluated at the Children’s Hospital of Eastern Ontario (CHEO) in Canada. Proband 2 had clinical exome sequencing performed through GeneDx. Proband 4 had chromosomal microarray analysis that was non-informative except for multiple regions of absence of heterozygosity (AOH) consistent with consanguinity in the family. Clinical exome sequencing for proband 4 was performed as a singleton through GeneDx.

Proband 3 was evaluated at Ochsner Medical Center in Jefferson, Louisiana, USA. He had uninformative chromosomal microarray analysis and fragile X testing. Clinical exome sequencing was performed through GeneDx.

Proband 5 was evaluated at Schneider’s Children Medical Center (SCMC) in Israel. Previous testing included normal 46XX karyotype and chromosomal microarray. Proband was also tested for Williams-Beuren Syndrome (WBS), which was negative. Research-based trio whole exome sequencing in the affected child and unaffected parents was performed in collaboration with the Regeneron Genetics Center (RGC) and analyzed as previously described [20].

Proband 6 was evaluated at Greenwood Genetic Center (GGC) in Greenwood, South Carolina, USA. Chromosomal microarray analysis was performed and showed a 440 kb gain variant of unknown significance (Table 1). This copy gain is within the common 15q13.3 micro-duplication/deletion region encompassing *CHRNA7*, mapping between breakpoints 4 and 5. Common 15q13.3 microduplications involving the *CHRNA7* gene are suggested to be of uncertain clinical significance [21] and incompletely penetrant [22]. Follow-up qPCR studies demonstrated maternal inheritance of this micro-duplication CNV. Other negative results included Rett and Prader-Willi/Angelman syndromes testing.

Proband 7 was evaluated at the Duke Institute for Brain Sciences (DIBS) in Durham, North Carolina, USA. Previous molecular testing included normal 46XY karyotype, muscular dystrophy panel, and Prader-Willi/Angelman syndrome FISH testing. Biochemical studies included normal amino acids, urine organic acids, lactate, pyruvate, and acylcarnitine profiles. Clinical exome sequencing was also performed through GeneDx.

### Exome sequencing and variant analysis methods

Proband 1 had clinical exome sequencing performed by Baylor Miraca Genetics Laboratories. The methodology employed for exome sequencing was as follows: genomic DNA was fragmented by sonication and ligated to Illumina multiplexing paired-end adapters. For exome target enrichment and capture, the pre-capture library was hybridized in solution to the VCRome 2.1 target probes. Sequencing was performed on the Illumina HiSeq platform for 100 bp paired-end reads to achieve at least >70% of reads aligned to target, and >95% of target bases covered at >20× with a mean coverage >100×. Sequence data was aligned to the GRCh37 (hg19) human genome reference sequence. Variants were interpreted according to the American College of Medical Genetics (ACMG) guidelines and patient phenotypes.

Probands 2, 3, 4, and 7 had clinical exome sequencing through GeneDx as follows. Using genomic DNA from the proband and parents (when available), the exonic regions and flanking splice junctions of the genome were captured using the SureSelect Human All Exon V4 (50 Mb), the Clinical Research Exome kit (Agilent Technologies, Santa Clara, CA) or the IDT xGen Exome Research Panel v1.0. Massively parallel (NextGen) sequencing was done on an Illumina system with 100 bp or greater paired-end reads. Reads were aligned to human genome build GRCh37/UCSC hg19 and analyzed for sequence variants using a custom-developed analysis tool. Additional sequencing technology and variant interpretation protocol has been previously described [23]. The general assertion criteria for variant classification are publicly available on the GeneDx ClinVar submission page (http://www.ncbi.nlm.nih.gov/clinvar/submitters/26957/). Candidate variants were confirmed by Sanger dideoxynucleotide sequencing.

For patient 5, exome capture was performed using the IDT xGen reagent (Integrated DNA Technologies, Coralville, IA, USA) and sequenced on the Illumina HiSeq 2500 platform using v4 chemistry (Illumina, San Diego, CA, USA) to achieve a minimum of 85% of the target bases covered at 20× or greater coverage. Sequence data was mapped and aligned to the human genome reference assembly GRCh38 (hg38). Called variants were annotated for their functional effects and predictions, conservation, and allele frequency in public and internal population databases. Standard quality control filters were applied for read depth (≥10×), genotype quality (≥30), and allelic balance (≥20%). Variants were filtered to exclude common, likely benign variants present in population databases (MAF ≤ 1%) and prioritized based on functional effect, deleteriousness potential, and inheritance pattern and segregation.

For patient 6, clinical whole exome sequencing was performed using the SureSelect^XT^ Clinical Research Exome (CRE) capture kit (Agilent Technologies, Santa Clara, CA) and sequenced using an Illumina NextSeq 500^®^ Sequencing System (Illumina Inc., San Diego, CA) per the manufacturer’s protocol. Sequences were processed using NextGENe software (SoftGenetics, LLC, State College, PA), and mapped to the February 2009 human reference assembly (GRCh37/hg19). Variants in the coding regions of the reference (and within 25 bp flanking) were retained if they had a read depth of >3× and a variant allele frequency of ≥20%. Variants were filtered and analyzed using the Cartagenia Bench Lab NGS software (Agilent Technologies, Santa Clara, CA). Variants not in the Human Genome Mutation Database (QIAGEN, Germantown, MD) were filtered based on population frequency in public SNP databases, functional effect prediction, and HPO phenotype. Quality and allele frequency filters were used to isolate variants with at least 20× read depth and 22% minor allele frequency. Candidate variants were confirmed by Sanger dideoxynucleotide sequencing.

All identified variants reported here have been deposited under submission ID SUB5492409 (accession numbers SCV000927107 through SCV000927118) for public access through ClinVar (https://www.ncbi.nlm.nih.gov/clinvar/).

## Results

Through a combination of genomic sequencing studies, community data sharing [24], and multi-institutional collaboration, we have identified and characterized the largest cohort of patients with biallelic disease causing variants in the *PTPN23* gene. The major clinical features in these patients are summarized below and in Table 1.

**Fig. 1:**
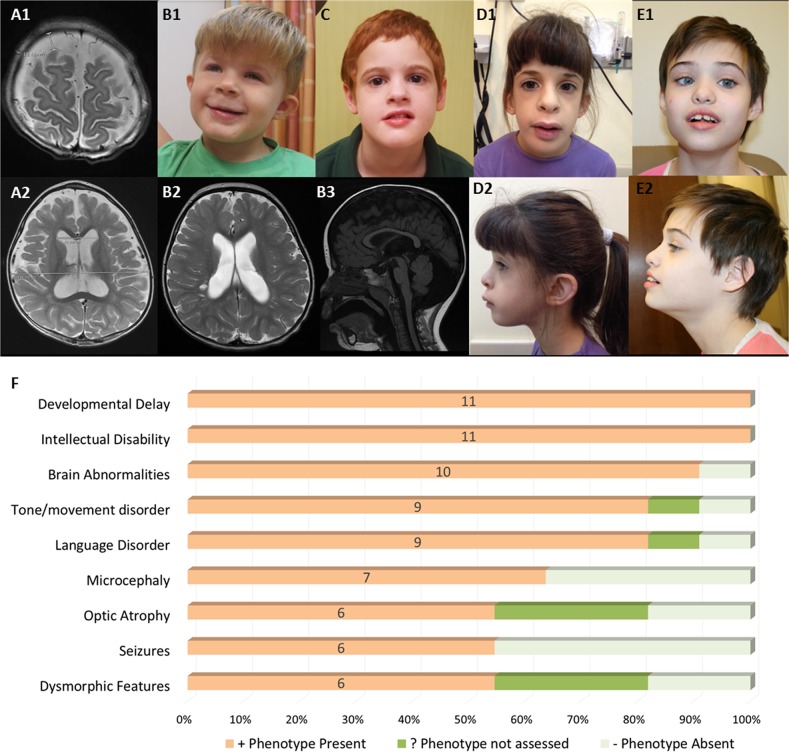
Photographs, MRI images, and phenotypes of patients with biallelic protein altering variants in *PTPN23*. Brain MRI images of Patient 1 (**a1** and **a2**) at 14 months of age showing mild ventriculomegaly and decrease in cerebral volume. Brain MRI images of Patient 2 (**b2** and **b3**) showing ventriculomegaly and brain volume loss. Patient brain abnormalities include ventriculomegaly, cerebral atrophy, abnormal myelination, and hypoplastic corpus callosum. Facial features observed in patients with biallelic variants in *PTPN23*: Patient 2 (**b1**), Patient 3 (**c**), Patient 5 (**d1** and **d2**), Patient 6 (**e1** and **e2**) included strabismus, abnormal nose and ear morphology, and broad mouth with full lips. Panel **f** shows the major phenotypic features identified in patients with biallelic disease causing variants in *PTPN23* including developmental delay and intellectual disability (100%), brain abnormalities (90.9%), mostly characterized by brain atrophy with cerebral volume loss, ventriculomegaly, and delayed myelination; tone or movement disorder, mainly spasticity (81.8%); language absence or delay (81.8%), microcephaly (63.6%), optic atrophy (54.5%), seizures (54.5%), and dysmorphic features (54.5%)

Proband 1 is a male child that presented for clinical evaluation at CNMC at 14 months of age due to developmental and motor delay. Gross and fine motor skills at 12.5 months corrected age were at 9 months. Brain MRI showed mild to moderate ventriculomegaly and enlarged subarachnoid spaces with communicating external hydrocephalus and mild decrease in cerebral volume; myelination was observed to be appropriate for age (Fig. 1A1 and A2). Proband also showed dysmorphic features including a flat occiput with brachycephaly, metopic ridging, left esotropia, arched eyebrows, and mild micrognathia. He had deep palmar creases with palmar pads, tracheoesophageal fistula, and mild left pelviectasis. WES analyses identified compound heterozygous missense variants (NM_015466.4:c.[2680 C > T];[2747 A > G], p.[(His894Tyr)];[(Gln916Arg)]) in *PTPN23*. Sanger confirmation and segregation showed that he had inherited the p.(His894Tyr) variant from his unaffected heterozygous father and the p.(Gln916Arg) variant from his unaffected heterozygous mother (Supplementary Material). Although these two missense variants appear to have low functional prediction scores using common in silico algorithms, both variants have high conservation scores in vertebrates and are very rare in available population cohorts (Table 2). The p.(His894Tyr) variant has been observed in 1 heterozygous individual in gnomAD (MAF = 0.000004206), whereas the p.(Gln916Arg) substitution has been observed in 8 heterozygotes in gnomAD (MAF = 0.00003382), with no reported homozygotes for either variant. Comparison of the clinical and radiological features, namely ventriculomegaly and decreased cerebral volume, in Proband 1 with other individuals identified with disease causing variants in *PTPN23* in this report and in the literature further strengthened the likely diagnosis of *PTPN23*-associated disorder. However, the possibility of an alternative diagnosis cannot be completely excluded until further experimental evidence is provided for the *PTPN23* variants identified.

**Table 2:** Genomic coordinates and annotation of *PTPN23* variants identified in patients from this cohort

Patient	Chr	Start	End	Ref	Alt	Zyg	Gene	Accession	Exon	rsID	Variant	GERP	LRT_PRED	SIFT_PRED	PROVEAN_PRED	PPH2_PRED	MTT_PRED	CADD	gnomAD FREQ	gnomAD Carriers	Inheritance
Patient 1	3	47410478	47410478	C	T	het	*PTPN23*	NM_015466	exon20	rs967738491	c.2680 C > T; p.His894Tyr	Conserved	Deleterious	Tolerated	Neutral	Benign	Polymorphism	0.256	0	HET = 0/ HOM = 0	Paternal
	3	47410545	47410545	A	G	het	*PTPN23*	NM_015466	exon20	rs770692989	c.2747 A > G; p.Gln916Arg	Conserved	Neutral	Tolerated	Neutral	Benign	Polymorphism	0.024	3.38E−05	HET = 8/ HOM = 0	Maternal
Patient 2	3	47410676	47410688	CAGCCCCATCCT	–	het	*PTPN23*	NM_015466	exon20	–	c.2878_2889del12; p.Gln960_Pro963del	NA	NA	NA	Deleterious	NA	Disease_causing	NA	0	HET = 0/ HOM = 0	Maternal
	3	47411546	47411546	G	A	het	*PTPN23*	NM_015466	exon20	–	c.3748 G > A; p.Glu1250Lys	Conserved	Deleterious	Tolerated	Neutral	Damaging	Disease_causing	21.8	7.45E−05	HET = 21/ HOM = 0	Paternal
Patient 3	3	47408451	47408451	C	T	het	*PTPN23*	NM_015466	exon15	rs150712932	c.1291 C > T; p.Arg431Trp	Conserved	Deleterious	Deleterious	Deleterious	Damaging	Disease_causing	25.3	1.42E−04	HET = 0/ HOM = 0	Maternal
	3	47410284	47410284	C	T	het	*PTPN23*	NM_015466	exon20	rs138076291	c.2486 C > T; p.Pro829Leu	Conserved	Neutral	Deleterious	Deleterious	Benign	Disease_causing	23.2	8.37E−06	HET = 2/ HOM = 0	Paternal
Patient 4	3	47410366	47410392	AGTTGCAGGTCTCCCCTCGGCCCCACC	–	hom	*PTPN23*	NM_015466	exon20	–	c.2568_2594del27; p.Val857_Pro865del	NA	NA	NA	Deleterious	NA	Disease_causing	NA	0	HET = 0/ HOM = 0	Maternal/ Paternal
Patient 5	3	47411682	47411684	AGA	–	hom	*PTPN23*	NM_015466	exon20	–	c.3884_3886del; p.1295_1296delLys	NA	NA	NA	Deleterious	NA	Disease_causing	NA	5.72E−04	HET = 159/ HOM = 0	Maternal/ Paternal
Patient 6	3	47412926	47412926	–	CC	hom	*PTPN23*	NM_015466	exon25	–	c.4651_4652dup; p.Leu1552Hisfs*33	NA	NA	NA	NA	NA	Disease_causing	NA	0	HET = 0/ HOM = 0	Maternal/ Paternal
Patient 7	3	47406548	47406548	G	A	het	*PTPN23*	NM_015466	exon8	rs577689618	c.695 G > A; p.Arg232Gln	Conserved	Deleterious	Deleterious	Neutral	Damaging	Disease_causing	24.1	3.89E−05	HET = 11/ HOM = 0	Paternal
	3	47409268	47409268	A	G	het	*PTPN23*	NM_015466	exon17	rs147293860	c.1748A > G; p.Lys583Arg	Conserved	Deleterious	Deleterious	Neutral	Damaging	Polymorphism	22.9	7.43E−05	HET = 21/ HOM = 0	Maternal
	3	47410849	47410849	G	C	het	*PTPN23*	NM_015466	exon20	rs201017613	c.3051 G > C; p.Gln1017His	Neutral	Neutral	Tolerated	Neutral	Benign	Disease_causing	10.8	2.48E−04	HET = 67/ HOM = 1	Maternal

**Fig. 2:**
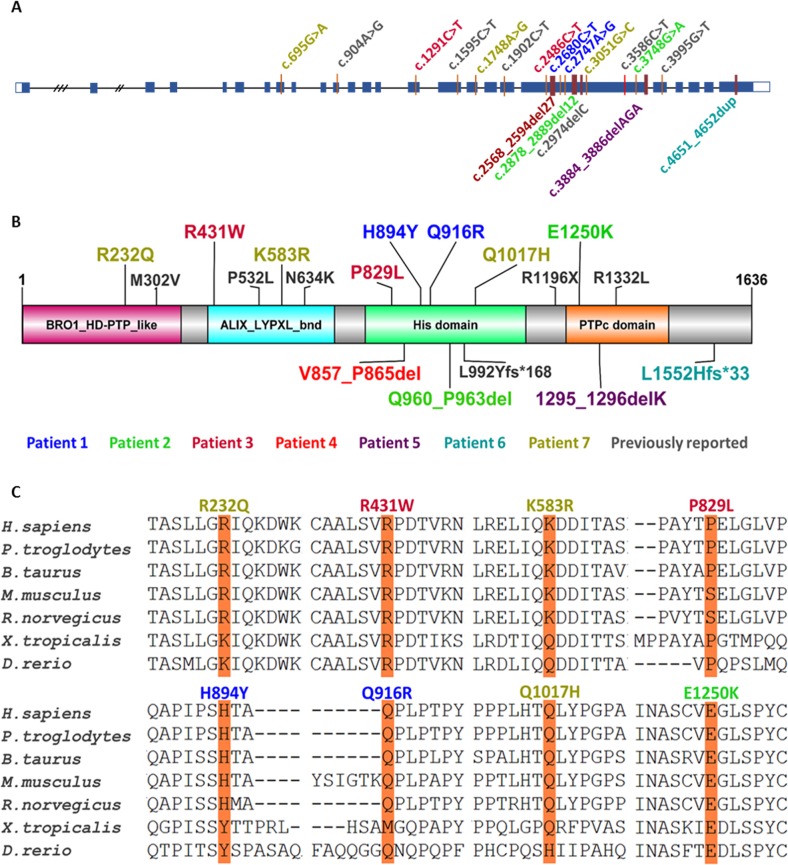
Variation spectrum identified in patients with biallelic rare protein altering variants in *PTPN23*. Variants are color coded according to described patients (Patients 1–7) in this report. Variants in dark gray are previously reported. Nonsynonymous variants are above the transcript (Panel **a**) and protein (Panel **b**) depictions; in-frame and frameshifting indel variants are below the transcript and protein depictions. The functional domains of PTPN23 are labeled (Panel **b**): The BRO1 HD-PTP like domain at the N-terminus that binds CHMP4B (charged multivesicular body protein 4B), an ALIX LYPXL binding domain, the His domain and the inactive Protein Tyrosine Phosphatase (PTP) C-terminal domain. Panel **c** shows the multiple species alignment and conservation of the residues altered by missense variants identified in patients with *PTPN23* associated neurodevelopmental disorder

Proband 2 is a male child evaluated at 2.5 years of age at CHEO. He was late to walk independently (~18 months) and remains very clumsy with bilateral intention tremor and shuffling gait at times, but not wide based. Speech was delayed with about 30 words at time of evaluation. He had an abnormal EEG (frequent isolated right parietal spike discharges) without overt seizures. Brain MRI performed at 2 years of age showed diffuse enlargement of the cerebral sulci and ventricular system which was suspicious for brain volume loss with prominent perivascular spaces (Fig. 1B2 and B3). He was short for his age, albeit not significantly (height: 89.5 cm; 15%, *Z* = −1.03). He had a prominent forehead and his palpebral fissures appeared subjectively short, but they were not formally measured. He had a prominent nasal root and a smooth philtrum with a thin upper lip, and low set ears that are somewhat simple, protruding, and cupped (Fig. 1B1). Hands and feet were unremarkable. He has a hypopigmented area ~4 cm in diameter on the left side of his neck that is best observed with a Woods lamp. Cardiorespiratory exam was normal. At 4.5 years of age he attends speech and occupational therapy at CHEO. Reportedly, his tremor significantly impacts his ability to feed himself and perform other fine motor tasks. Speech development has not improved significantly despite intensive therapy; he uses an iPad with communication software to indicate his basic needs. He does not appear to have any visual complaints; however no formal evaluation has been performed by an ophthalmologist. He has not developed seizures. Clinical WES identified compound heterozygous variants in *PTPN23*, a missense (NM_015466.4:c.3748 G > A, p.(Glu1250Lys)) variant inherited from his unaffected father and an in-frame deletion (NM_015466.4:c.2878_2889del12, p.(Gln960_Pro963del)) that was inherited from his unaffected mother (Supplementary Material). The maternally inherited in-frame deletion variant is novel and has not been previously reported, while the missense p.(Glu1250Lys) variant inherited from the father has been observed in 21 heterozygotes in gnomAD but no homozygotes (MAF = 0.00007454) (Table 2). This missense variant affects a highly conserved amino acid residue and is predicted to be deleterious by multiple bioinformatic algorithms (Fig. 2, Table 2).

Proband 3 is a male individual evaluated at Ochsner Medical Center at 6 years of age. He presented with developmental delay, intellectual disability, autism spectrum disorder, and apraxia. He had a history of ventricular septal defect and patent ductus arteriosus. He was noted to have speech dyspraxia, hyperlexia, and a sensory processing disorder. He is mildly dysmorphic with synophrys, a broad and flat nasal bridge, and full lips (Fig. 1c). He had an unremarkable brain MRI and unremarkable selective spectroscopy of the brain. Clinical exome sequencing identified compound heterozygous missense variants in *PTPN23* (NM_015466.4:c.[1291 C > T];[2486 C > T], p.[(Arg431Trp)];[(Pro829Leu)]). Sanger sequencing and segregation in the proband and both parents showed that the variants were in *trans* in the proband, having inherited the p.(Arg431Trp) variant from his unaffected heterozygous mother and the p.(Pro829Leu) from his unaffected father (Table 2, Supplementary Material).

Proband 4 was a 7-year-old female from a consanguineous Syrian family. Family history was significant for a similarly affected brother who died at 6 years of age in Syria. She had severe growth restriction; at 7 years OFC was 47 cm (−3SD), length was not measurable due to contractures, and weight was 11.1 kg (−4SD). She had spastic quadriparesis, scoliosis, and multiple large and small joint contractures. She was confined to a wheelchair with little purposeful movement and no head control. She had cataracts and significant visual impairment with abnormal eye movements and poor pupillary reactions to light. She had not attained language skills. She also had a history of recurrent infections and aspiration pneumonia. Dysmorphic features were notable for a long and narrow face with very prominent ears. Brain MRI was performed in Syria and results were unavailable, but parents recalled findings consistent with atrophy and possible lissencephaly. The patient passed away at 7 years of age. Exome sequencing revealed a homozygous in-frame variant in *PTPN23* (NM_015466.4:c.2568_2594del27; p.(Val857_Pro865del)) that was further confirmed by Sanger sequencing (Supplementary Material). This in-frame deletion of nine amino acids of PTPN23 is novel and has not been observed in the heterozygous or homozygous state in any publicly available databases. Seven of the nine deleted amino acids are highly conserved in mammals and the variant is predicted to be deleterious for protein function. Of note, another rare homozygous variant of unknown significance in *EOMES* (NM_001278182.1:c.692 A > G; p.(Tyr231Cys)) was identified in linkage disequilibrium with the *PTPN23* variant within the same region of homozygosity.

Proband 5 is a 10-year-old female from an Ashkenazi Jewish family from Israel. She was evaluated at SCMC due to developmental delay and mild intellectual disability. She has difficulty with social interactions. Brain MRI showed insular and parietoocipital polymicrogyria with partial agenesis of the corpus callosum and hypoplasia of the optic nerves. She was noted to be different in appearance to her parents with dysmorphic features including a long face, prominent forehead, malar hypoplasia, a long and smooth philtrum, a broad and long mouth with full lips, hypoplasia of ala nasi, anteverted nares, bulbous nasal tip, low-hanging collumela, posteriorly rotated ears, and narrow and high-arched palate (Fig. 1 D1 and D2). She had fetal fat pads and *pes planus*, but no skeletal abnormalities. Trio WES analysis identified a homozygous in-frame deletion of one amino acid variant (NM_015466.4: c.3884_3886delAGA; p.(1295_1296delLys)) in *PTPN23* as the main candidate for the phenotype in this proband. Although this variant is an in-frame deletion of one amino acid, multiple species alignment shows that this Lysine at position 1296 of PTPN23 is extremely well-conserved, suggesting it might be relevant for the function of the protein. This variant has been previously reported and observed in 159 heterozygous individuals in gnomAD (MAF = 0.0005721) but no homozygotes. This variant is most prevalent in Ashkenazi Jewish individuals (MAF = 0.01273) consistent with the reported ancestry of this patient and her parents.

Proband 6 is a female patient of European ancestry with no reported parental consanguinity. She was evaluated at GGC due to developmental delay with regression at 2 years of age and carried a diagnosis of cerebral palsy. She is nonverbal at 11 years of age. She has microcephaly with delayed myelination and enlargement of the posterior left lateral ventricle, optic atrophy, and optic nerve hypoplasia observed on MRI. She also has spastic diplegia, hypotonia, contractures at the knees, femoral anteversion that was surgically corrected, slow gait with bent knees, and hyperreflexia with multiple beats of clonus at the ankles. Other features noted were constipation and past sleep problems that were resolved. EEG was normal and no seizure disorder was noted or reported. She was mildly dysmorphic with strabismus, thin eyebrows, prominent and wide-spaced upper incisors, broad mouth and full lips (Fig. 1E1 and E2). Narrow hands and feet were also noted. A homozygous frameshift variant (NM_015466.4:c.4651_4652dup; p.(Leu1552Hisfs*33)) in the last exon was identified in *PTPN23* through trio exome sequencing. Because of its location, this frameshift variant in the last exon is expected, and further predicted through in silico methods [25], to escape nonsense mediated decay and result in a truncated protein product. Both parents were confirmed to be heterozygous carriers for this rare variant, which has not been reported elsewhere. Of note, this rare variant in *PTPN23* was found to be embedded within a 14.3 Mb region of absence of heterozygosity (AOH) at 3p21.31p14.2 (Table 1). Total autosomal AOH for the proband was only 0.8% consistent with reported non-consanguinity of the parents; however, it is suggestive of the haplotype carrying this variant being identical by-descent in both parents.

Proband 7 is a 13-year-old Hispanic male evaluated at DIBS. He has a history of developmental delay and intellectual disability with bilateral brain atrophy observed on MRI. He has short stature, hypotonia, areflexia, neuromuscular scoliosis, and joint contractures. He had precocious puberty, central sleep apnea, and expressive language delay. He remains nonverbal. He has a history of intractable seizures that started at 2 years of age and complicated by apnea with insomnia and frequent sleep arousals. He is not particularly dysmorphic but does have strabismus. WES analysis identified three rare missense variants in *PTPN23* in this proband. Two variants (NM_015466.4:c.[1748A > G; 3051 G > C], p.[(Lys583Arg); p.(Gln1017His)]) are in *cis* and were inherited from his unaffected mother. Both maternally inherited variants are rare, and neither could be definitely excluded; however, the p.(Gln1017His) variant is more frequent (MAF = 0.0002481) and has been observed in one homozygous individual in gnomAD. The residue being altered is also less well-conserved and the substitution is predicted to be more tolerated. Conversely, the p.(Lys583Arg) has been observed in 21 heterozygous individuals but no homozygotes in gnomAD (MAF = 0.00007427), the affected residue is highly conserved across species, and the amino acid change is predicted to be deleterious (Table 2). The proband also inherited in *trans* a missense (NM_015466.4:c.695 G > A; p.(Arg232Gln)) variant from his unaffected heterozygous father. This variant is also present in gnomAD (MAF = 0.00003892) but with no reported homozygous instances (Table 2).

## Discussion

The main clinical findings and features for the patients reported here and in the four previously reported patients are summarized in Table 1 and Fig. 1. Overall, we observe some major features shared among the majority of patients with biallelic protein altering variants in *PTPN23*, namely developmental delay (100%), intellectual disability (100%), brain abnormalities (91%), mostly characterized by brain atrophy with cerebral volume loss, ventriculomegaly, and delayed myelination; tone or movement disorder (82%), language absence or delay (82%), microcephaly (63%), and optic atrophy (54%). Interestingly, although seizures were one of the main findings in the initially reported patients, in our cohort this is not a consistent feature; only 2 of the 7 patients in our cohort were reported to have a history of febrile or intractable seizures, with an overall 54% of all patients reported to date having epileptic encephalopathy. Therefore, epilepsy should not be considered a cardinal clinical sign for the diagnosis of this neurodevelopmental disorder. Dysmorphic features were observed among patients including prominent forehead, deep set eyes, strabismus, prominent ears, broad and flat nasal bridge with a broad nose tip, and a broad mouth with full lips.

*PTPN23* is located in chromosome 3p21.3 and is composed of 24 exons. The variation spectrum that we observe in our cohort of patients includes missense (*N* = 8), frameshift (*N* = 1), and in-frame deletion variants (*N* = 3) (Fig. 2). The identified missense variants occur in highly conserved residues supporting their candidacy for affecting PTPN23 function (Fig. 2c). We do not observe a clear correlation between identified protein altering variants and disease severity across our patients and the reported cases. However, some of the homozygous individuals, such as Patient 4 in this report and the first patient reported by Alazami et al. [14], and individuals with one predicted loss-of-function allele in *trans* of another non-truncating allele appear to be more severely affected. Furthermore, although in some cases compound heterozygosity for predicted loss-of-function, either nonsense or frameshift variants, in *trans* with rare predicted deleterious missense variants has been observed, no homozygous or compound heterozygous individuals with biallelic predicted loss-of-function variants have yet been reported by us or others. The only homozygous frameshift variant (NM_015466.4:c.[4651_4652dup]; p.(Leu1552Hisfs*33)) identified to date in our Patient 6 occurs in the last exon of the gene and it is predicted to escape nonsense mediated decay producing a smaller truncated protein product lacking the last 51 amino acids, with an aberrant C-terminal region. The absence of patients reported so far with biallelic loss-of-function variants would be consistent with the early embryonic lethality reported in homozygous knockout mice and therefore suggests that the rare missense and in-frame deletion variants identified in patients are likely hypomorphic alleles that may retain some functional activity of PTPN23 protein. Characterization of these variants at the molecular and biochemical level will help elucidate the precise functional effects on the protein. For example, Sowada et al. [16] performed in silico modeling of the reported variants in PTPN23 and propose that the p.(Pro532Leu) variant likely alters the structure of the protein reducing its stability and affecting its sorting function. The p.(Arg1332Leu) variant is also predicted to disrupt the structure of the protein, primarily of the PTPase domain, by destabilizing the molecular interactions of this domain with other structurally relevant amino acids and with its substrates. Finally, the p.(Met302Val) variant is predicted to have a milder effect, mostly reducing protein stability and potentially altering binding to other proteins and protein complexes [16]. More recently, Smigiel et al. [17] functionally studied fibroblasts derived from their patient, carrying a predicted protein truncating variant in *trans* with a rare p.(Asn634Lys) missense variant. While they observed expression of the PTPN23 protein, they reported strongly reduced SMN accumulation in Cajal bodies versus increased nucleoplasmic levels of SMN in the patient derived cells compared to unaffected parent and control fibroblasts, in spite of similar SMN protein expression [17]. Altogether, these analyses and observations support the hypothesis that disease associated variants that affect *PTPN23* function are likely hypomorphic and suggest that residual PTPN23 function is necessary for human viability and early development; whereas, similarly to mouse, complete loss of function due to biallelic truncating variants in *PTPN23* is likely lethal in humans.

Smigiel et al. suggest abnormal SNM phosphorylation and localization or impaired maturation of uridine-rich small nuclear ribonucleoproteins (UsnRNPs) as possible mechanisms of disease for *PTPN23* protein altering variants [17]. However, the multiple processes in which PTPN23 has been implicated suggest that defects in this gene can lead to the observed phenotype through abnormal processing and trafficking of a wider variety of protein complexes, in addition to SMN. For example, impaired ESCRT function has been associated with intracellular accumulation of ubiquitinated proteins that may aggregate in the brain and cause neuronal degeneration and microcephaly [26, 27]. In addition, *PTPN23* has been nominated as a tumor suppressor candidate gene due to its apparent function in regulating cell migration and possibly autophagy [8]. These functions could very well play a role in the structural brain abnormalities and microcephaly observed in individuals with biallelic variants in this gene that affect correct protein function.

While four patients had previously been identified and reported in the literature, the work presented here details the genetic and phenotypic analyses of the largest series of patients with biallelic disease causing variants in *PTPN23*, enabling characterization of this novel genetic disorder. The majority of variants identified in patients with this genetic disorder are rare or have not previously been observed in population databases. However, as exemplified by Proband 5, some variants may be rare founder alleles in certain populations, such as Ashkenazi Jewish individuals, and should be considered in screening panels for intellectual disability. We have clinically characterized seven patients and expanded the phenotypic and disease associated variation spectrum of the *PTPN23* associated disorder characterized by developmental delay, intellectual disability, brain abnormalities including ventriculomegaly and brain atrophy, optic nerve abnormalities, spasticity, and variable seizures. Identification and characterization of these seven patients in the context of the previously reported patients help to further identify major clinical features and define the phenotypic spectrum of the disease. Once recognized molecularly, genotype-phenotype correlation and identification of shared clinical features can enable more accurate clinical diagnoses of this rare disorder and inform natural history of the disease for future patients.

## Supplementary information

PTPN23_Cohort_Supplemental_Material
