# Supplementary material for: Phenotype and mutation expansion of the PTPN23 associated disorder characterized by neurodevelopmental delay and structural brain abnormalities
Source: Eur J Hum Genet. 2019 Aug 8;28(1):76–87. doi: 10.1038/s41431-019-0487-1 (PMC6906308; doi:10.1038/s41431-019-0487-1)
Supplement: Supplementary file 1 — PTPN23_Cohort_Supplemental_Material [file 41431_2019_487_MOESM1_ESM.pdf]

Phenotype and mutation spectrum expansion of the *PTPN23* associated disorder characterized by neurodevelopmental delay and structural brain abnormalities

Renee Bend, Lior Cohen, Melissa T. Carter, Michael J. Lyons, Dmitriy Niyazov, Mohamad A. Mikati, Samantha K. Rojas, Richard E. Person, Yue Si, Ingrid M. Wentzensen, Regeneron Genetics Center, Erin Torti, Jennifer A. Lee, Kym M. Boycott, Lina Basel-Salmon, Claudia Gonzaga-Jauregui, Carlos R. Ferreira

Supplementary Material

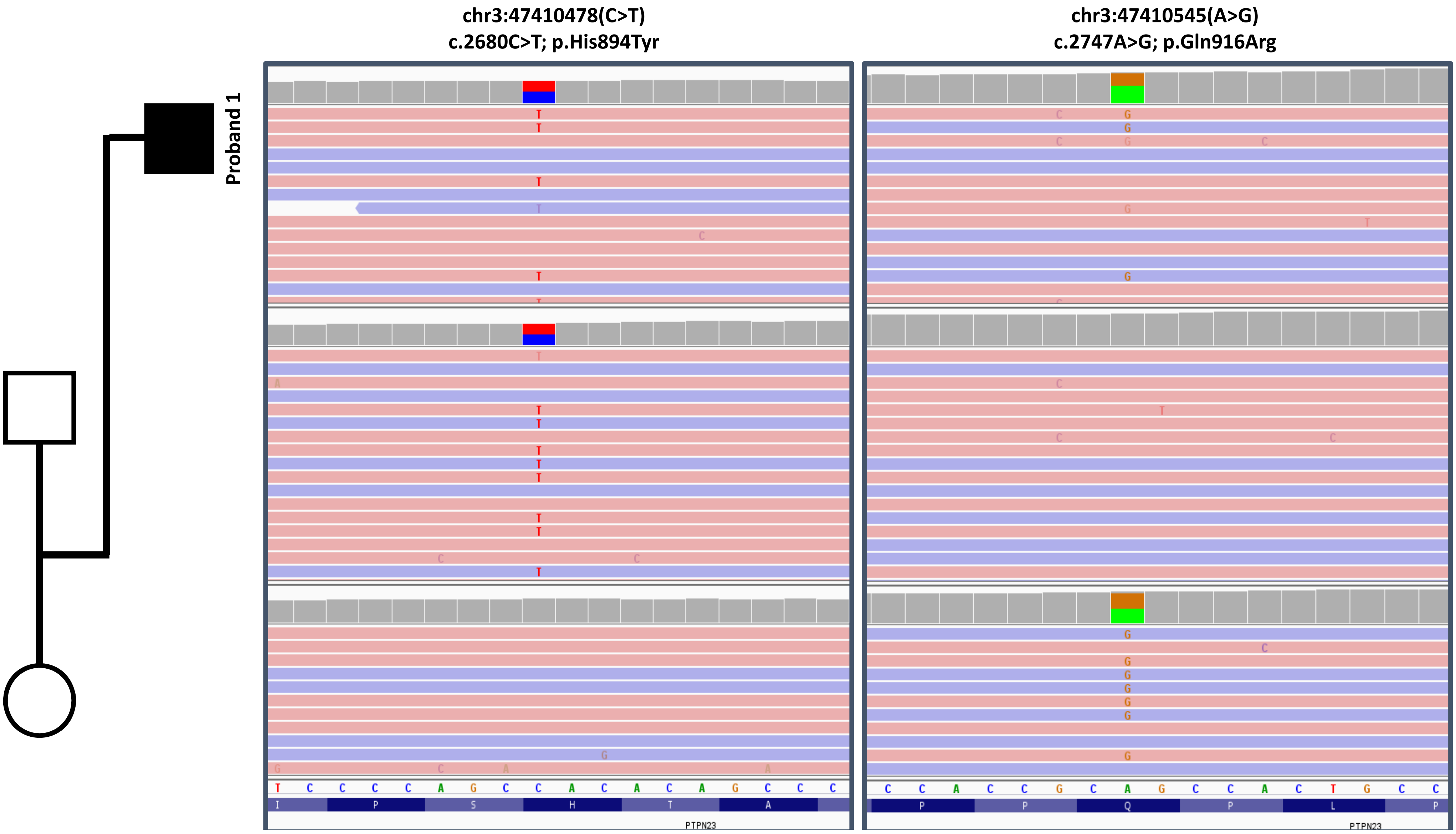
chr3:47410478(C>T)
c.2680C>T; p.His894Tyr
chr3:47410545(A>G)
c.2747A>G; p.Gln916Arg
Proband 1
PTPN23
PTPN23

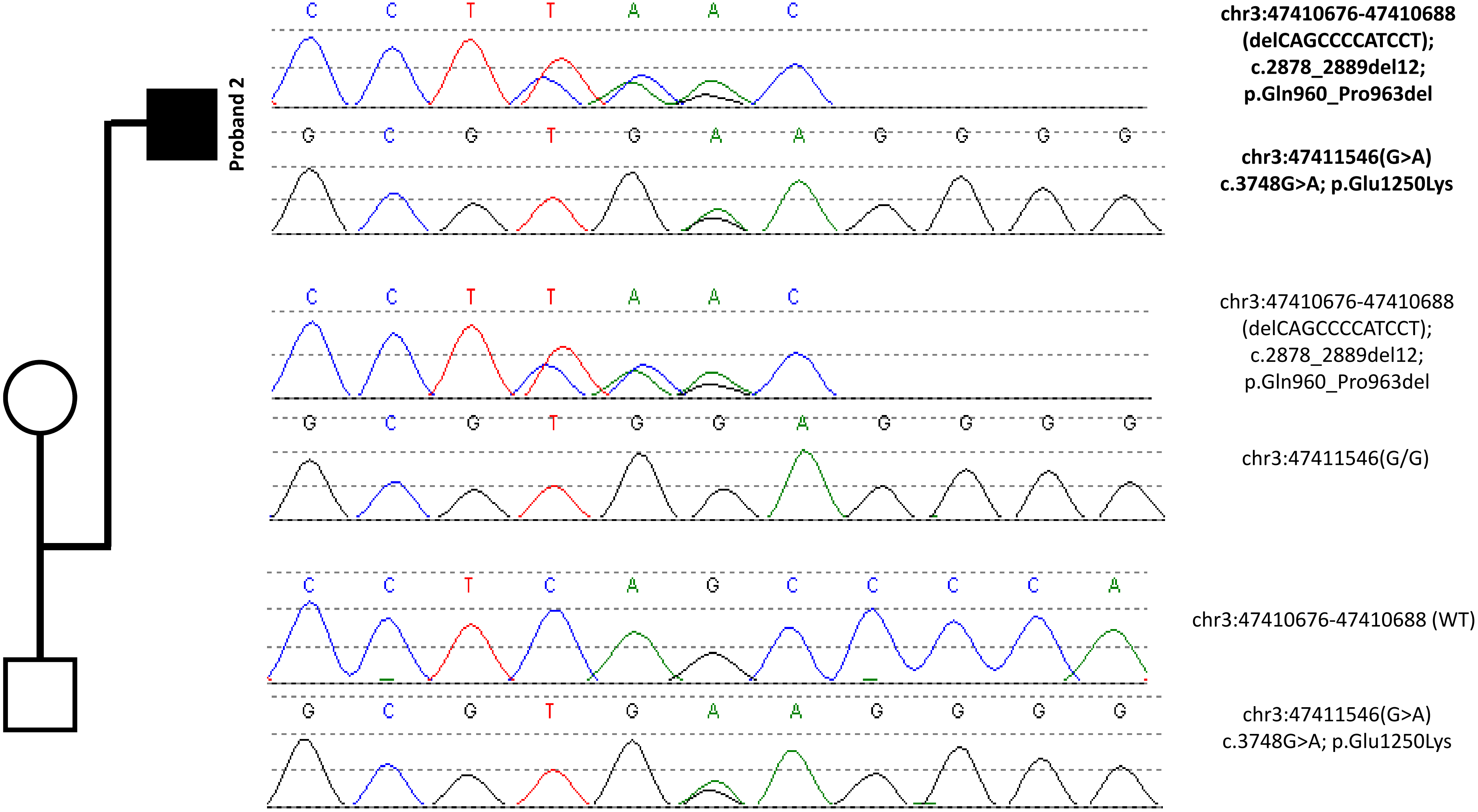
Proband 2
C C T T A A C
G C G T G A A G G G G
chr3:47410676-47410688
(delCAGCCCCATCCT);
c.2878_2889del12;
p.Gln960_Pro963del
chr3:47411546(G>A)
c.3748G>A; p.Glu1250Lys
C C T T A A C
G C G T G G A G G G G
chr3:47410676-47410688
(delCAGCCCCATCCT);
c.2878_2889del12;
p.Gln960_Pro963del
chr3:47411546(G/G)
C C T C A G C C C C A
G C G T G A A G G G G
chr3:47410676-47410688 (WT)
chr3:47411546(G>A)
c.3748G>A; p.Glu1250Lys

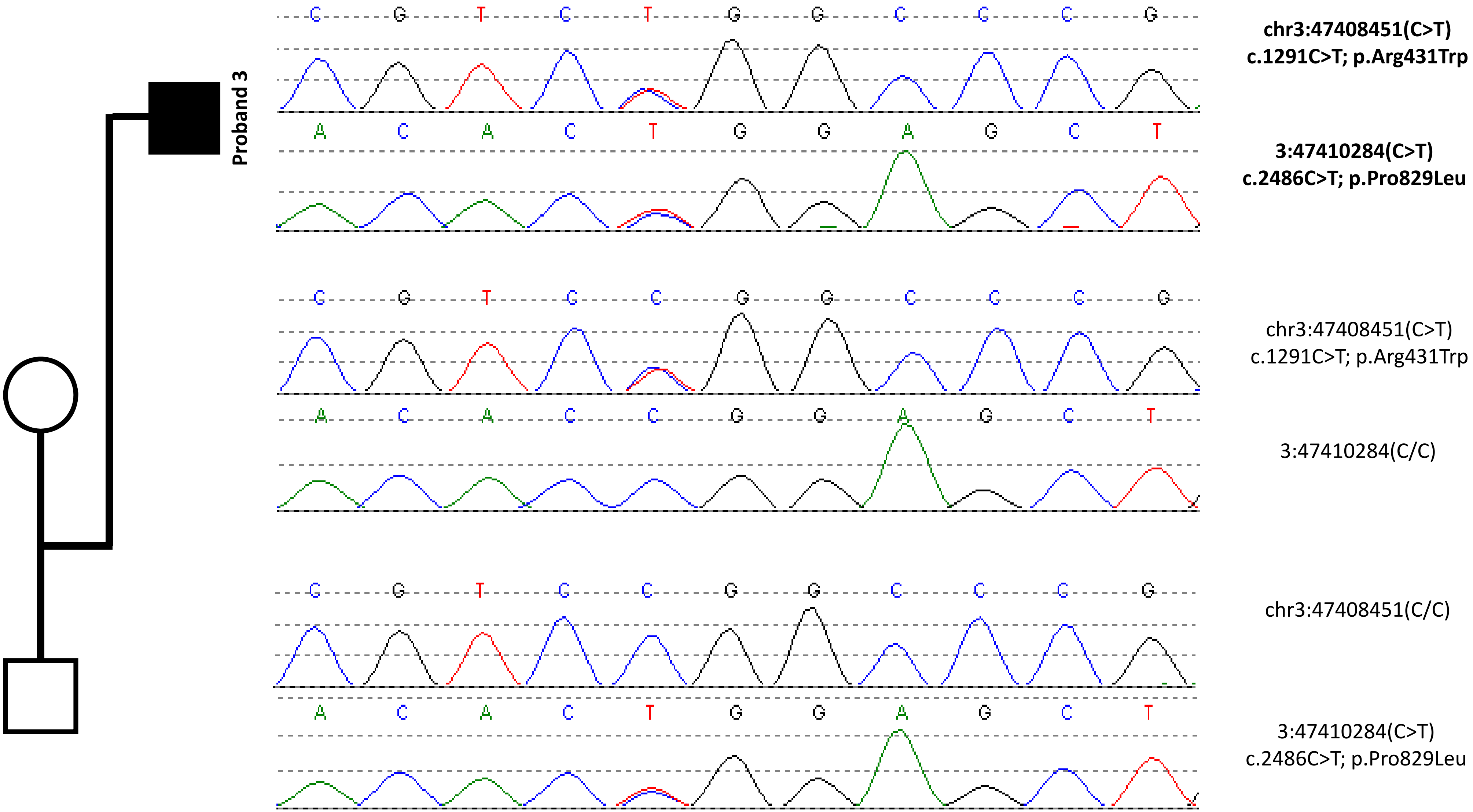

Proband 3
chr3:47408451(C>T)
c.1291C>T; p.Arg431Trp
3:47410284(C>T)
c.2486C>T; p.Pro829Leu
chr3:47408451(C>T)
c.1291C>T; p.Arg431Trp
3:47410284(C/C)
chr3:47408451(C/C)
3:47410284(C>T)
c.2486C>T; p.Pro829Leu

chr3:47410366-47410392 (delAGTTGCAGGTCTCCCCTCGGCCCCACC); c.2568_2594del27; p.Val857_Pro865del

Proband 4

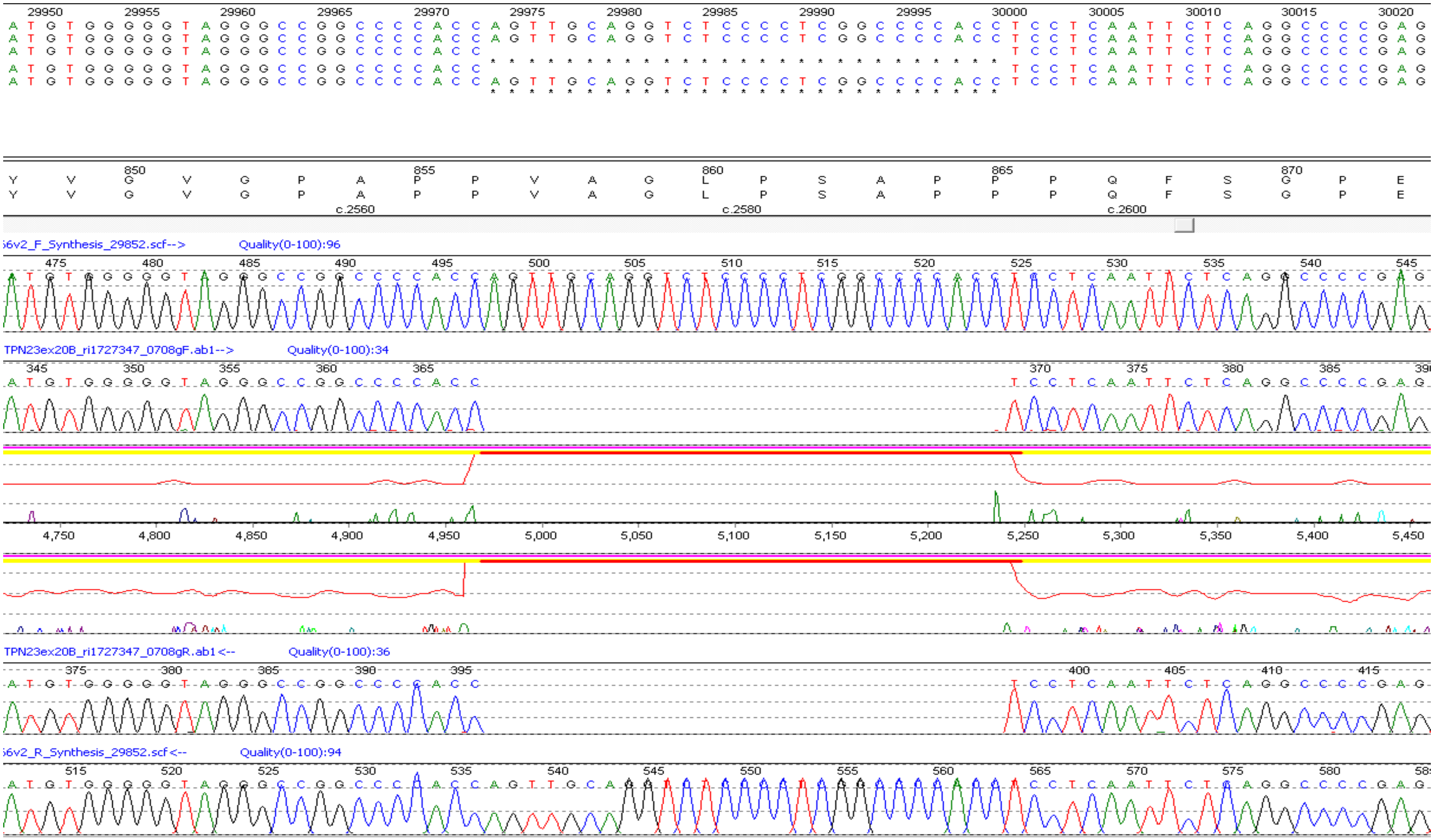

chr3:47411682-47411684(delAGA); c.3884_3886del; p.1295_1296delLys

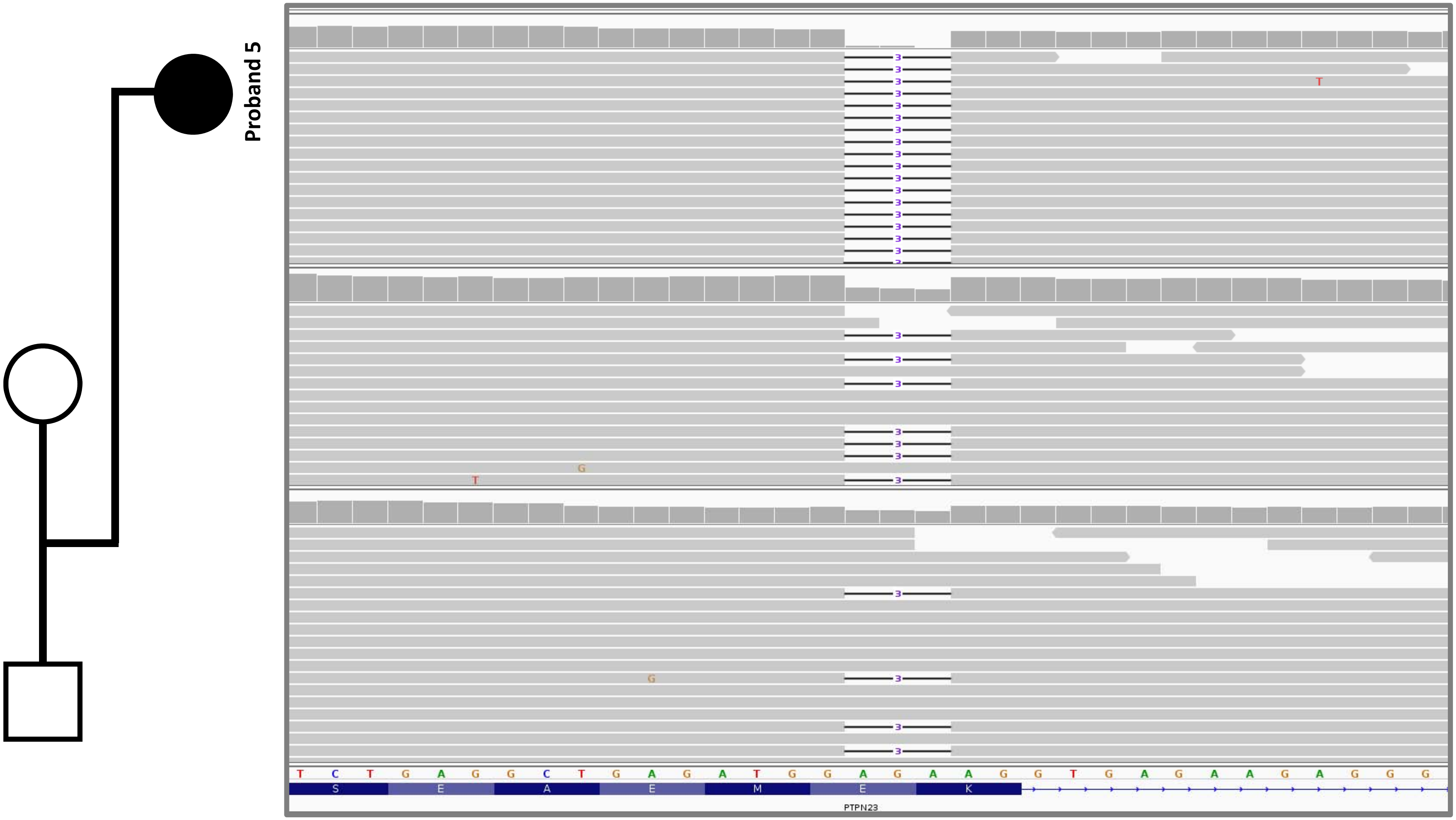

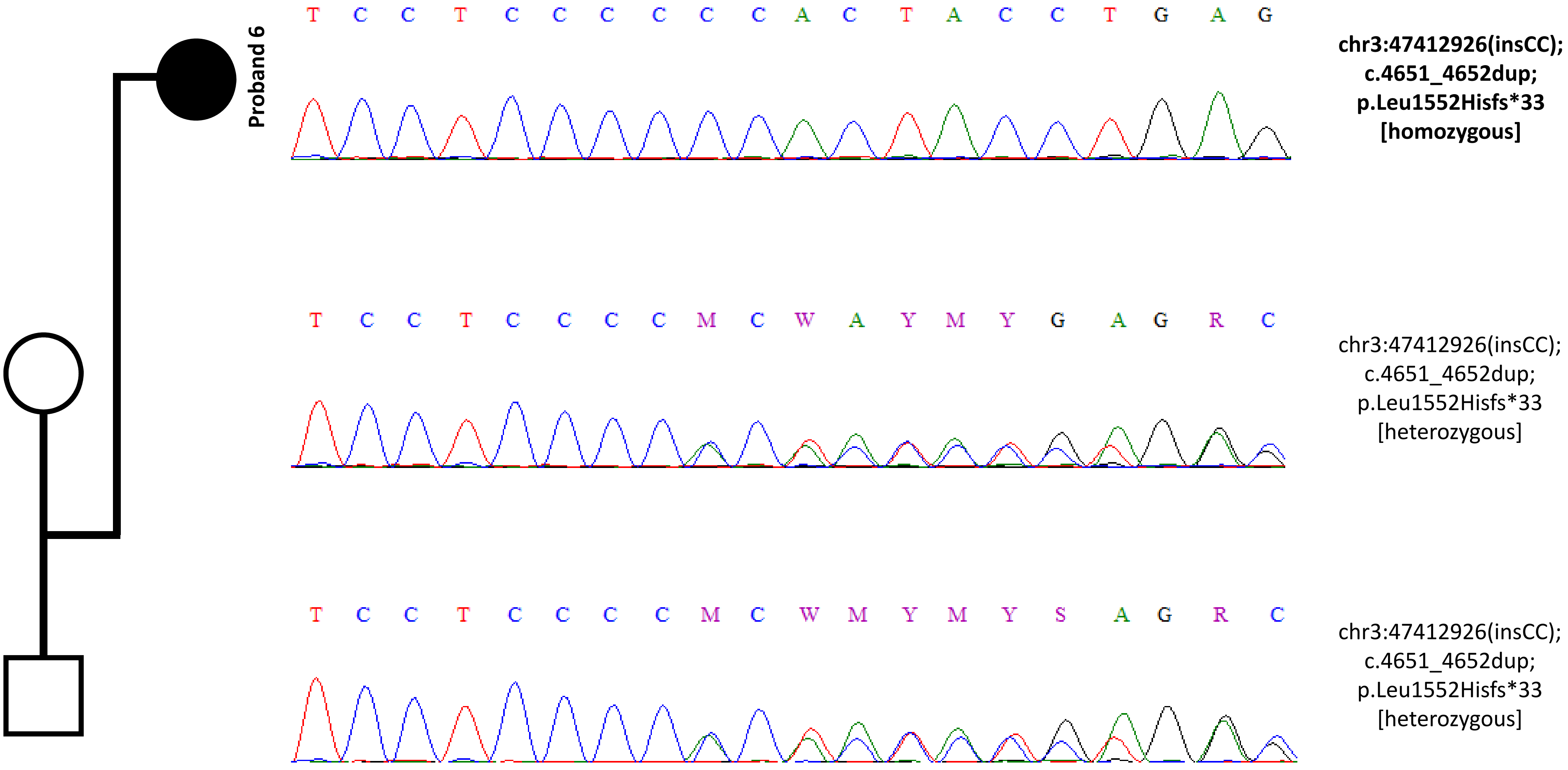

Proband 6
T C C T C C C C C C A C T A C C T G A G
chr3:47412926(insCC);
c.4651_4652dup;
p.Leu1552Hisfs*33
[homozygous]
T C C T C C C C M C W A Y M Y G A G R C
chr3:47412926(insCC);
c.4651_4652dup;
p.Leu1552Hisfs*33
[heterozygous]
T C C T C C C C M C W M Y M Y S A G R C
chr3:47412926(insCC);
c.4651_4652dup;
p.Leu1552Hisfs*33
[heterozygous]

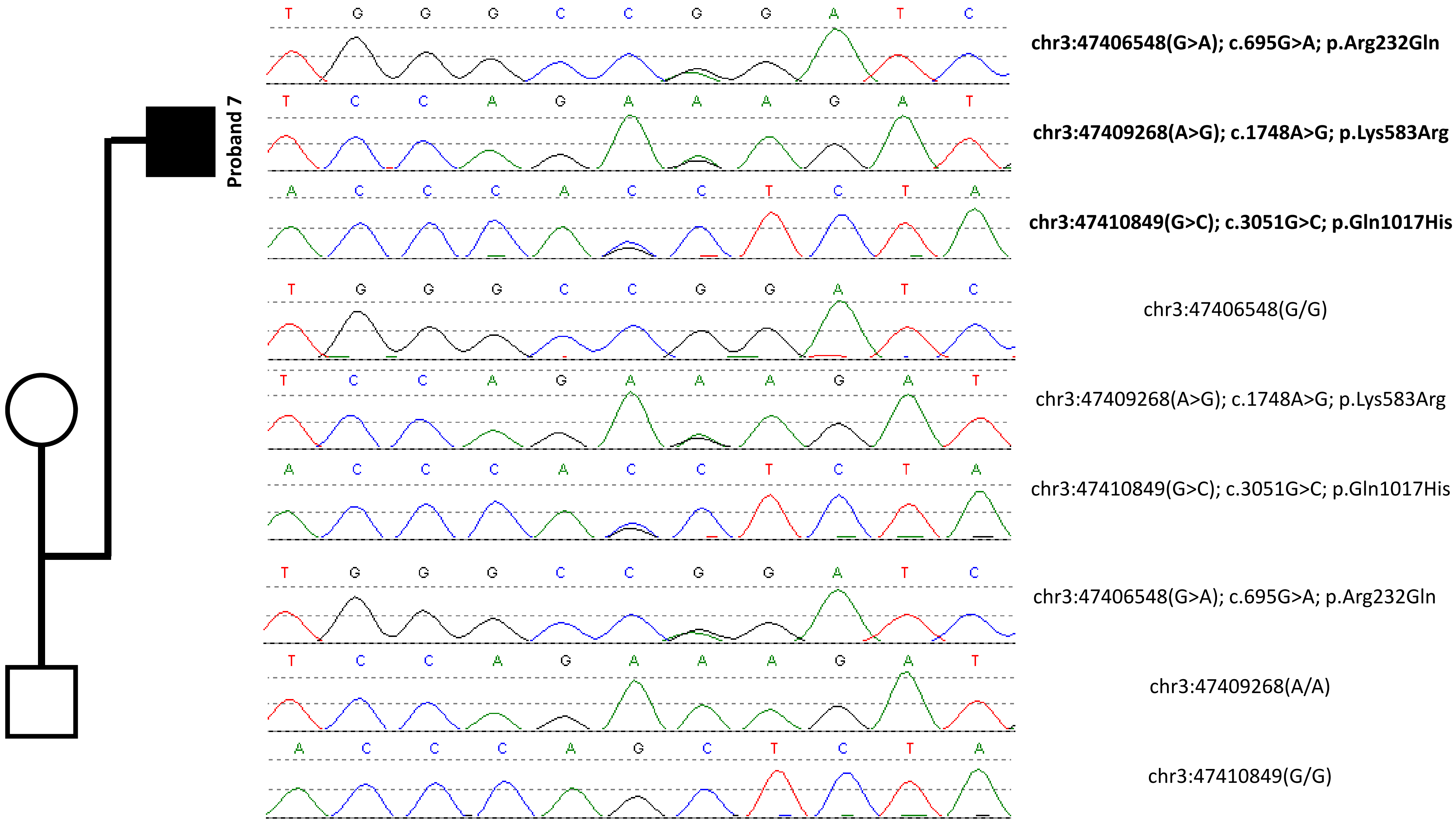
Proband 7
T G G G C C G G A T C
chr3:47406548(G>A); c.695G>A; p.Arg232Gln
T C C A G A A A G A T
chr3:47409268(A>G); c.1748A>G; p.Lys583Arg
A C C C A C C T C T A
chr3:47410849(G>C); c.3051G>C; p.Gln1017His
T G G G C C G G A T C
chr3:47406548(G/G)
T C C A G A A A G A T
chr3:47409268(A>G); c.1748A>G; p.Lys583Arg
A C C C A C C T C T A
chr3:47410849(G>C); c.3051G>C; p.Gln1017His
T G G G C C G G A T C
chr3:47406548(G>A); c.695G>A; p.Arg232Gln
T C C A G A A A G A T
chr3:47409268(A/A)
A C C C A G C T C T A
chr3:47410849(G/G)
